# A second dose of kisspeptin-54 improves oocyte maturation in women at high risk of ovarian hyperstimulation syndrome: a Phase 2 randomized controlled trial

**DOI:** 10.1093/humrep/dex253

**Published:** 2017-08-08

**Authors:** Ali Abbara, Sophie Clarke, Rumana Islam, Julia K Prague, Alexander N Comninos, Shakunthala Narayanaswamy, Deborah Papadopoulou, Rachel Roberts, Chioma Izzi-Engbeaya, Risheka Ratnasabapathy, Alexander Nesbitt, Sunitha Vimalesvaran, Rehan Salim, Stuart A Lavery, Stephen R Bloom, Les Huson, Geoffrey H Trew, Waljit S Dhillo

**Affiliations:** 1 Department of Investigative Medicine, Imperial College London, Hammersmith Hospital Campus, Du Cane Road, London, W12 0NN, UK; 2 IVF Unit, Hammersmith Hospital, Du Cane Road, London, W12 0HS, UK; 3 Division of Experimental Medicine, Hammersmith Hospital, Du Cane Road, London, W12 0HS, UK

**Keywords:** IVF, ICSI outcome, OHSS, oocyte maturation, kisspeptin, trigger injection

## Abstract

**STUDY QUESTION:**

Can increasing the duration of LH-exposure with a second dose of kisspeptin-54 improve oocyte maturation in women at high risk of ovarian hyperstimulation syndrome (OHSS)?

**SUMMARY ANSWER:**

A second dose of kisspeptin-54 at 10 h following the first improves oocyte yield in women at high risk of OHSS.

**WHAT IS KNOWN ALREADY:**

Kisspeptin acts at the hypothalamus to stimulate the release of an endogenous pool of GnRH from the hypothalamus. We have previously reported that a single dose of kisspeptin-54 results in an LH-surge of ~12–14 h duration, which safely triggers oocyte maturation in women at high risk of OHSS.

**STUDY DESIGN, SIZE, DURATION:**

Phase-2 randomized placebo-controlled trial of 62 women at high risk of OHSS recruited between August 2015 and May 2016. Following controlled ovarian stimulation, all patients (*n* = 62) received a subcutaneous injection of kisspeptin-54 (9.6 nmol/kg) 36 h prior to oocyte retrieval. Patients were randomized 1:1 to receive either a second dose of kisspeptin-54 (D; Double, *n* = 31), or saline (S; Single, *n* = 31) 10 h thereafter. Patients, embryologists, and IVF clinicians remained blinded to the dosing allocation.

**PARTICIPANTS/MATERIALS, SETTING, METHODS:**

*Study participants*: Sixty-two women aged 18–34 years at high risk of OHSS (antral follicle count ≥23 or anti-Mullerian hormone level ≥40 pmol/L).

*Setting*: Single centre study carried out at Hammersmith Hospital IVF unit, London, UK.

*Primary outcome*: Proportion of patients achieving an oocyte yield (percentage of mature oocytes retrieved from follicles ≥14 mm on morning of first kisspeptin-54 trigger administration) of at least 60%.

*Secondary outcomes*: Reproductive hormone levels, implantation rate and OHSS occurrence.

**MAIN RESULTS AND THE ROLE OF CHANCE:**

A second dose of kisspeptin-54 at 10 h following the first induced further LH-secretion at 4 h after administration. A higher proportion of patients achieved an oocyte yield ≥60% following a second dose of kisspeptin-54 (Single: 14/31, 45%, Double: 21/31, 71%; absolute difference +26%, CI 2–50%, *P* = 0.042).

Patients receiving two doses of kisspeptin-54 had a variable LH-response following the second kisspeptin dose, which appeared to be dependent on the LH-response following the first kisspeptin injection. Patients who had a lower LH-rise following the first dose of kisspeptin had a more substantial ‘rescue’ LH-response following the second dose of kisspeptin. The variable LH-response following the second dose of kisspeptin resulted in a greater proportion of patients achieving an oocyte yield ≥60%, but without also increasing the frequency of ovarian over-response and moderate OHSS (Single: 1/31, 3.2%, Double: 0/31, 0%).

**LIMITATIONS, REASONS FOR CAUTION:**

Further studies are warranted to directly compare kisspeptin-54 to more established triggers of oocyte maturation.

**WIDER IMPLICATIONS OF THE FINDINGS:**

Triggering final oocyte maturation with kisspeptin is a novel therapeutic option to enable the use of fresh embryo transfer even in the woman at high risk of OHSS.

**STUDY FUNDING/COMPETING INTEREST(S):**

The study was designed, conducted, analysed and reported entirely by the authors. The Medical Research Council (MRC), Wellcome Trust & National Institute of Health Research (NIHR) provided research funding to carry out the studies. There are no competing interests to declare.

**TRIAL REGISTRATION NUMBER:**

Clinicaltrial.gov identifier NCT01667406

**TRIAL REGISTRATION DATE:**

8 August 2012.

**DATE OF FIRST PATIENT'S ENROLMENT:**

10 August 2015.

## Introduction

Infertility is an involuntary disability afflicting one in six couples, resulting in devastating psychological distress (Demyttenaere *et al.*, 1998). Whilst IVF treatment is increasingly used to help such couples to conceive, it can be complicated by the potentially life-threatening complication ovarian hyperstimulation syndrome (OHSS) (Delvinge and Rozenberg, 2002; Thomsen and Humaidan, 2015; Toftager *et al.*, 2016). Of 387 399 cycles carried out in Europe in 2012 (for whom complete data was available), 7.1% of cycles started were cancelled prior to oocyte retrieval and 19.4% of cycles started did not have a fresh embryo transfer (Calhaz-Jorge *et al.*, 2016). The most frequent reasons cited for cycle cancellation prior to embryo transfer were either due to an insufficient response to stimulation, or due to concern of ovarian over-response and OHSS (Human Fertilisation and Embryology Authority, 2016). Thus, there remains a clinical need to improve triggering of oocyte maturation to optimize both the efficacy and safety of IVF treatment.

Many aspects of the IVF protocols used to achieve controlled ovarian stimulation, oocyte maturation and embryo formation aim to simulate the physiological processes observed in the normal human menstrual cycle. One of the major causes of OHSS is the use of hCG to trigger oocyte maturation during IVF treatment (Delvinge and Rozenberg, 2002; Thomsen and Humaidan, 2015; Toftager *et al.*, 2016). hCG is an effective trigger of oocyte maturation, but its pharmacokinetic properties (*t*_1/2_ 38 h) (Ezcurra *et al.*, 2014) result in a prolonged duration of action at the LH receptor (>1 week) in contrast to the physiological LH-surge which has a plateaux lasting 24–28 h (Hoff *et al.*, 1983; Castillo *et al.*, 2014). As such, hCG can cause moderate OHSS in 10.2% and severe OHSS in a further 5.1% of patients undergoing IVF treatment (Toftager *et al.*, 2016). Moreover, the risk of severe OHSS is further increased five-fold in patients with risk factors such as polycystic ovarian syndrome (PCOS) (Wada *et al.*, 1993) to as much as a quarter of patients (Seyhan *et al.*, 2013). Furthermore, late pregnancy complications such as prematurity and low birth weight are more frequently observed in women affected by OHSS in early pregnancy (Courbiere *et al.*, 2011; Haas *et al.*, 2014). GnRH-agonists are a safer alternative than hCG to trigger oocyte maturation, but are often used as second-line agents due to concerns regarding implantation rates (Youssef *et al.*, 2011). In addition, a number of case reports have suggested that severe OHSS may still occur in the high risk patient even when triggered with a GnRH-agonist and with segmentation (Fatemi *et al.*, 2014; Gurbuz *et al.*, 2014; Ling *et al.*, 2014).

Kisspeptin-54 is a key regulator of the human reproductive axis (Clarke and Dhillo, 2016). Kisspeptin signalling is requisite for the normal initiation of puberty (de Roux *et al.*, 2003; Seminara *et al.*, 2003) and is obligatory for physiological ovulation in animals (Matsui *et al.*, 2004; Caraty *et al.*, 2007; Clarkson *et al.*, 2008). We have recently reported that a single injection of kisspeptin-54 resulted in a luteinizing hormone (LH) surge of ~12–14 h duration, which was sufficient to safely trigger oocyte maturation in women at high risk of OHSS (Abbara *et al.*, 2015). Oocyte maturation (at least one mature oocyte) was observed in 95% of patients following kisspeptin-54 triggering (Abbara *et al.*, 2015); nevertheless, it is also important to consider the ‘oocyte yield’ achieved. Oocyte yield denotes the proportion of mature oocytes retrieved from follicles of sufficient size (≥14 mm) to yield an oocyte. Patients achieving a good oocyte yield are more likely to progress to subsequent stages of IVF treatment. In our previous study, kisspeptin-54 (9.6 nmol/kg) was an efficacious trigger of oocyte maturation resulting in an overall oocyte yield of 86%, however there was variation between individuals in the oocyte yield achieved (SD 49%). It is increasingly recognized that the efficacy of therapies should be demonstrated at the level of the individual rather than across a group (Schork, 2015). Therefore, we aimed to investigate whether oocyte maturation could be optimized using kisspeptin-54 to ensure that a clinically effective oocyte yield (≥60%) was achieved in as many individuals as possible and thus minimize the risk of cycle cancelations due to suboptimal efficacy or safety concerns.

Kisspeptin-54 results in a shorter duration of LH-exposure than the physiological LH-surge (Hoff *et al.*, 1983; Jayasena *et al.*, 2014; Abbara *et al.*, 2015). Thus, we hypothesized that extending the duration of LH-exposure by administering a second dose of kisspeptin-54 10 h after the first could further optimize oocyte maturation and increase the proportion of women achieving an oocyte yield of at least 60%. To test this hypothesis, we carried out a Phase-2 placebo-controlled clinical trial randomizing 62 women at high risk of OHSS to receive either one or two doses of kisspeptin-54 to trigger oocyte maturation during IVF treatment.

## Materials and Methods

### Study approval

The study was approved by the Hammersmith and Queen Charlotte's Research Ethics Committee, London, UK (reference: 10/H0707/2) and was undertaken at the IVF Unit at Hammersmith Hospital under a licence from the UK Human Fertilization and Embryology Authority. All participants provided written informed consent prior to inclusion in the study.

The study was registered on the National Institutes of Health Clinical Trials database (NCT01667406) and performed in accordance with the Declaration of Helsinki.

### Study participants

Seventy-six women requiring IVF treatment for infertility at Hammersmith Hospital, London, UK, were screened for participation between August 2015 and May 2016. The inclusion criteria aimed to select women at high risk of OHSS: serum anti-Müllerian hormone (AMH) ≥40 pmol/L (≥5.6 ng/mL) or total antral follicle count (AFC) ≥23 (Lee *et al.*, 2008; Jayaprakasan *et al.*, 2012); age 18–34 years; early follicular phase serum follicle stimulating hormone (FSH) ≤12 iU/L; both ovaries intact; body mass index 18–29 kg/m^2^. Exclusion criteria were moderate/severe endometriosis and poor response to or ≥2 previous cycles of IVF treatment.

**Figure 1:**
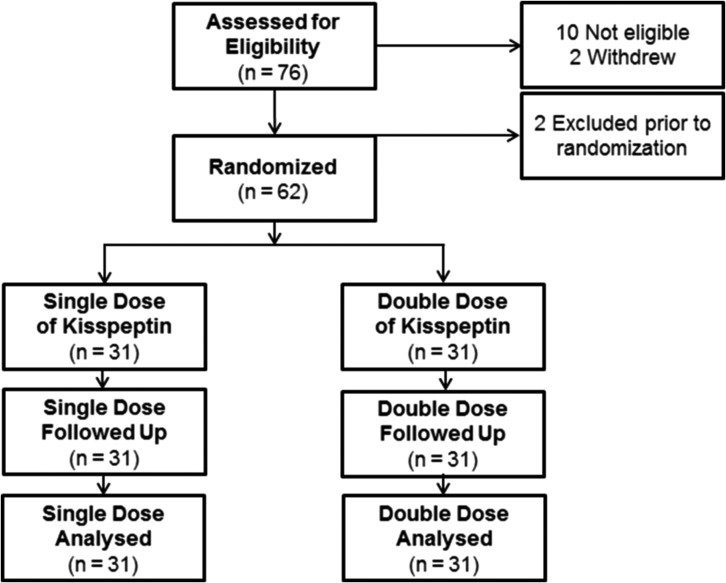
Patient flow diagram showing the number of patients assessed for eligibility, study enrolment and kisspeptin-54 dosing group allocation. Seventy-six patients were screened for participation in the study, of whom 62 women at high risk of ovarian hyperstimulation syndrome (OHSS) were randomized 1:1 to receive either one (Single, *n* = 31) or two (Double, *n* = 31) doses of kisspeptin-54 to trigger oocyte maturation.

Ten patients were not eligible for inclusion and two patients withdrew consent prior to commencing the study protocol (Fig. 1). A further two patients developed conditions requiring further management during the stimulation phase beyond the realms of the study protocol (e.g. hydrosalpinx) and were thus excluded prior to randomization. All treatment costs for the study cycle were covered by study participation. Sixty-two eligible patients underwent a single IVF treatment cycle and were randomized to receive either one (single; *n* = 31) or two doses (double; *n* = 31) of kisspeptin-54 to trigger oocyte maturation.

### Protocol

**Figure 2:**
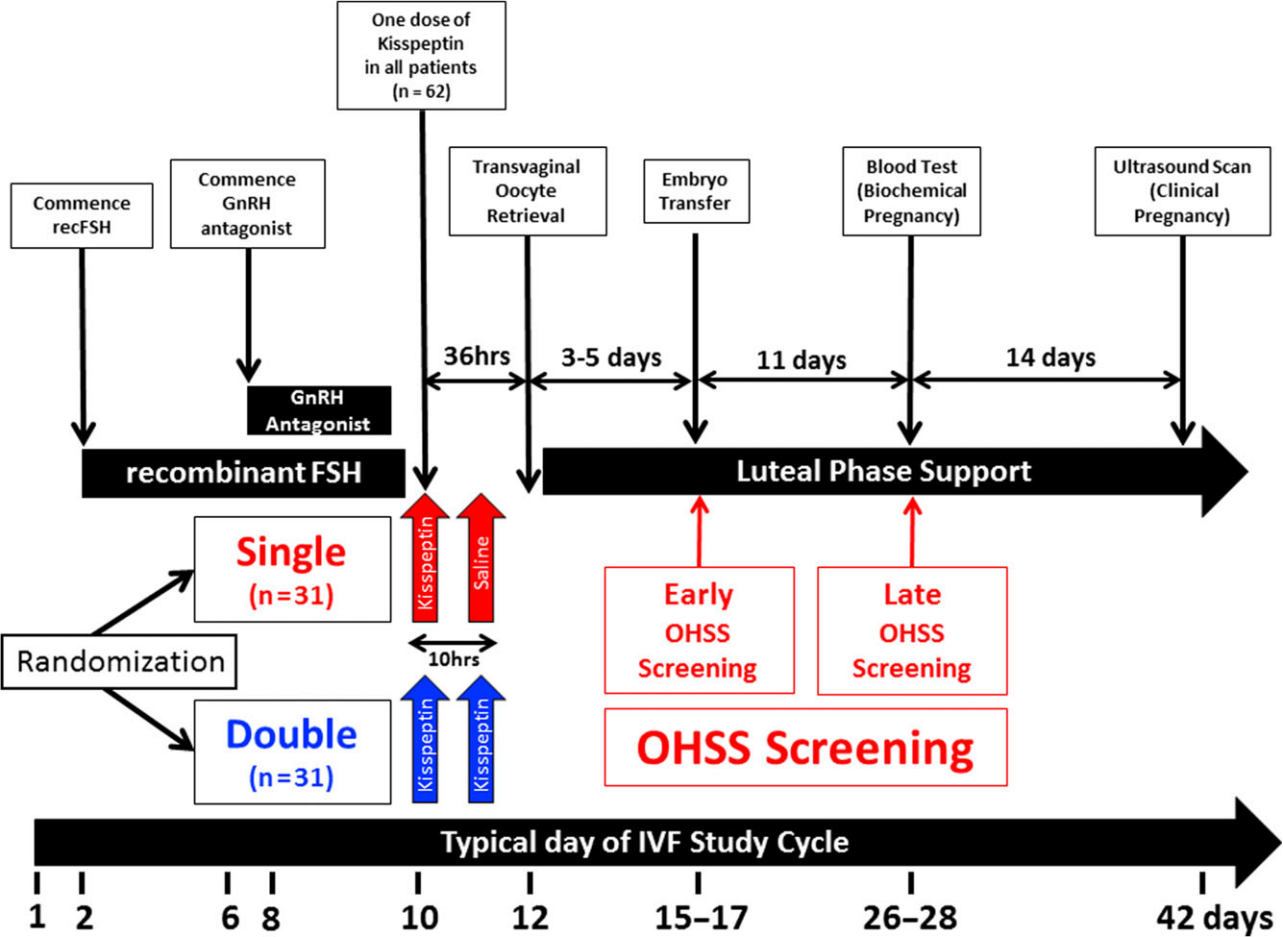
In vitro fertilization study protocol using kisspeptin-54 to trigger oocyte maturation. The timeline shows the day of menstrual cycle for a typical patient. On Day 2 or 3 of the menstrual cycle, daily subcutaneous recombinant FSH (Gonal F 112.5 IU) was commenced. Daily GnRH antagonist injections (Cetrotide 0.25 mg) were commenced after 5 days of recombinant FSH injections. If serum LH was undetectable (<0.5 IU/L) on Day 7 of recombinant FSH injections, the dose of cetrotide was halved to 0.125 mg daily. When at least three ovarian follicles ≥18 mm diameters were visible on ultrasound, all patients (*n* = 62) received a subcutaneous injection of kisspeptin-54 (9.6 nmol/kg) 36 h prior to oocyte retrieval to trigger oocyte maturation (between 20 :30 and 23:00 h). Injections of GnRH-antagonist and FSH were stopped 24 h and 12 h prior to the first kisspeptin-54 injection, respectively. We have previously shown that this protocol and dose of kisspeptin-54 safely and effectively triggers oocyte maturation in women at high risk of ovarian hyperstimulation syndrome (OHSS) undergoing IVF treatment (Abbara *et al.*, 2015). All patients then received a second injection 10 h after the first injection, but were randomized 1:1 to receive either a second dose of kisspeptin-54 (D; Double, *n* = 31), or saline (S; Single, *n* = 31) to determine if a second dose of kisspeptin-54 could increase the duration of LH-exposure and optimize oocyte yield compared to a single dose of kisspeptin-54. Serum reproductive hormones (LH, FSH, estradiol and progesterone) were measured immediately prior to as well as 4 and 10 h after the first kisspeptin-54 injection. Serum reproductive hormones were also measured immediately prior to as well as 4 and 10 h after the second injection (of kisspeptin-54 or saline). Transvaginal ultrasound-directed oocyte retrieval (TVOR) was carried out 36 h following the first kisspeptin-54 injection, and ICSI was performed using fresh sperm from the male partner. If a high quality blastocyst was available, elective single embryo transfer (eSET) was carried out 5 days following oocyte retrieval. Progesterone 100 mg daily intramuscular injections (Gestone, Nordic Pharma, UK) and estradiol valerate 2 mg orally three times daily (Progynova, Bayer, Germany) was commenced from the evening of TVOR until 12 weeks gestation. All women recruited to the study were regarded as being at high risk of OHSS and were routinely screened for the development of early OHSS (assessed on day of embryo transfer 3–5 days after TVOR) and late OHSS (assessed 11 days after embryo transfer). Biochemical pregnancy (serum βHCG > 10 iU/L) was assessed 11 days following embryo transfer and clinical pregnancy was assessed by ultrasonography at 6 weeks gestation.

Following a standard recombinant FSH/GnRH-antagonist IVF protocol, all patients (*n* = 62) received a subcutaneous injection of kisspeptin-54 (9.6 nmol/kg) 36 h prior to oocyte retrieval to trigger oocyte maturation once at least three follicles were ≥18 mm in diameter (further details of the protocol are presented in ‘Supplemental methods’). Patients were randomized 1:1 on the day of trigger to receive either a second dose of kisspeptin-54 (Double, *n* = 31), or saline (Single, *n* = 31) 10 h thereafter (see Fig. 2 for study protocol). Serum reproductive hormones (LH, FSH, estradiol and progesterone) were measured immediately prior to, as well as 4 and 10 h after the first kisspeptin-54 injection and at the same time-points relative to the second injection (kisspeptin-54 or saline). Elective single embryo transfer (eSET) was performed in all patients with at least one high quality blastocyst formed.

### Assessment of ovarian hyperstimulation syndrome

All women recruited to the study were regarded as being at high risk of OHSS and were routinely screened for the development of early OHSS (assessed at embryo transfer 3–5 days following oocyte retrieval) and late OHSS (assessed 11 days following embryo transfer) as previously described (Abbara *et al.*, 2015). In short, assessment for OHSS included symptomatic, sonographic and biochemical evaluation in accordance with published guidance for the reporting of OHSS in clinical trials (Humaidan *et al.*, 2016). Blinded screening data was independently graded by two experienced IVF clinicians external to the study team (S.L. and R.S.), according to the criteria of Golan *et al.* (1989) with updated categorization by Navot *et al.* (1992) (Navot *et al.*, 1992; Golan and Weissman, 2009). In the event of any discrepancy in categorization of OHSS, the more severe classification was reported.

Further details of the IVF protocol, embryo grading, peptide synthesis, OHSS screening and hormonal assay methodology have been published previously (Abbara *et al.*, 2015) and are detailed in ‘Supplemental methods’.

### Study outcomes

The primary outcome was the proportion of patients achieving an oocyte yield (percentage of mature oocytes collected from the number of follicles ≥14 mm in diameter on ultrasound scan performed on the morning of first kisspeptin-54 trigger administration) of at least 60%.

Secondary outcomes were reproductive hormone levels, implantation rate (proportion of embryos transferred that implant at 6 weeks gestation) and occurrence of OHSS.

### Randomization and masking

The number of patients assessed for eligibility, study enrolment and dose allocation are outlined in the CONSORT diagram in Fig. 1. Randomization was performed using printed lists prepared by the trial statistician (L.H.) using SAS 9.3. Sixty-two patients were randomized 1:1 to receive either one (single) or two (double) doses of kisspeptin-54 (31 patients per group) on the morning of triggering. All patients randomized were included in the analysis of primary and secondary outcomes on an intention-to-treat basis. A saline-placebo injection was administered to participants randomized to the ‘single’ kisspeptin-54 group such that participants, embryologists and IVF clinicians remained blinded to the dose allocation (‘single’ or ‘double’ dose of kisspeptin-54).

### Sample size and power

The sample size (31 patients per group) was calculated based on oocyte yield data from a previous clinical trial in patients at high risk of OHSS who received 9.6 nmol/kg of kisspeptin-54 to trigger oocyte maturation (Abbara *et al.*, 2015). Using Fisher's exact conditional test for two proportions, a sample size of 31 patients per group (±5% drop-out rate) was determined to provide an 80% power using a two-sided α of 0.05 to detect an expected increase in the proportion of patients achieving an oocyte yield ≥60%, rising from 43% with a single kisspeptin-54 injection to 80% with a double kisspeptin-54 injection.

### Statistical analysis

Data analysis was performed by L.H., who had no involvement in patient management or data collection. The primary endpoint (proportion of patients achieving an oocyte yield ≥60%) was compared between treatment groups using a logistic regression model. From the model, an estimate of the difference in success rates and an estimate of the odds ratio for success were derived, together with a *P* value comparing the two treatment groups, and 95% confidence intervals. Similar models and analyses were performed for the other two binary endpoints: implantation rate and clinical pregnancy rate. Relevant baseline covariates were tested in all of these models, but none were statistically significant, and so all covariates were excluded from these models for derivation of the final results.

## Results

Following a recombinant FSH/GnRH-antagonist IVF protocol, all patients (*n* = 62) received a subcutaneous injection of kisspeptin-54 (9.6 nmol/kg) 36 h prior to oocyte retrieval. Patients were randomized 1:1 to receive either a second dose of kisspeptin-54 (D; Double, *n* = 31), or saline (S; Single, *n* = 31) 10 h thereafter (Fig. 2).

### Baseline characteristics

Sixty-two women were randomized to receive either one (*n* = 31) or two doses of kisspeptin-54 (*n* = 31) (see Fig. 1 for CONSORT diagram). No imbalances in baseline characteristics were observed between the dosing groups (see Table I for baseline characteristics).

**Table I:** Baseline characteristics of patients who received kisspeptin-54 trigger.

*N*	Single	Double	Both
	31	31	62
Age (y)	31.2 (27.9, 33.4)	30.9 (29.1, 32.6)	31.1 (29, 32.7)
Weight (kg)	61.6 (58.4, 68.2)	68.1 (57.4, 76.1)	64.5 (58.4, 73)
Body mass index (kg/m^2^)	23.9 (21.9, 27.2)	26.6 (22.8, 28.9)	25.5 (22.7, 28)
Antral follicle count (AFC)	39 (30, 52)	33 (29, 40)	36 (29, 48)
Serum AMH^a^ (pmol/L)	52.4 (36, 81.4)	44.0 (34.6, 56.4)	45.7 (36, 63.5)
Number of patients with average cycle length ≥35 days number (%)	13 (41.9%)	11 (35.5%)	24 (38.7%)
*Cause of infertility*			
PCOS^b^	17 (54.8%)	18 (58.1%)	35 (56.5%)
Tubal	1 (3.2%)	0 (0.0%)	1 (1.6%)
Male Factor	4 (12.9%)	2 (6.5%)	6 (9.7%)
Mixed	1 (3.2%)	1 (3.2%)	2 (3.2%)
Idiopathic (unexplained)	8 (25.8%)	10 (32.3%)	18 (29.0%)
Number of follicles^c^	35.0 (25.0, 44.0)	31.0 (25.0, 41.0)	33.5 (25.0, 42.0)
Number of follicles ≥11 mm^c^	24.0 (16.0, 31.0)	23.0 (15.0, 28.0)	23.5 (15.0, 30.0)
Number of follicles ≥14 mm^c^	17.0 (10.0, 22.0)	14.0 (11.0, 21.0)	16.0 (11.0, 21.0)
Serum estradiol on day of trigger (pmol/L)	12 712 (8333, 19 471)	11 256 (7716, 16 744)	11 414 (8295, 17 986)
Number of patients with serum estradiol on day of trigger ≥11 000 pmol/L (%)	22 (71.0%)	24 (77.4%)	46 (74.1%)

Contains medians (lower quartile, upper quartile) for continuous variables and numbers of patients (percentages) for categorical variables. Single = patients receiving a single injection of kisspeptin. Double = patients receiving a second injection of kisspeptin 10 h after the first. Both = data for both single and double groups combined.

^a^AMH = serum anti-Müllerian hormone in pmol/L.

^b^Anovulation due to polycystic ovarian syndrome.

^c^On final ultrasound scan during controlled ovarian stimulation on morning of first kisspeptin-trigger.

### Oocyte maturation

Oocyte maturation (at least one mature oocyte) was observed in the majority of patients following kisspeptin-54 trigger (61/62; 98.4%): almost all patients (30/31; 96.8%) in the single group and all patients in the double group (31/31; 100%) had at least one mature oocyte retrieved and fertilized to form an embryo for transfer. In our study, 3 of 31 (9.7%) patients in the single group, but none of the double group had fewer than four oocytes retrieved (see Table II for further detail on markers of oocyte maturation by kisspeptin-54 dosing group and ‘Supplemental Figure S1’ for histogram of number of oocytes retrieved by kisspeptin-54 dosing group).

**Table II:** Primary and secondary outcomes with additional clinical parameters for IVF following kisspeptin-54 trigger.

	Single (*n* = 31)	Double (*n* = 31)	AD (CI, *P* value)	Both (*n* = 62)
Primary outcome: number of patients achieving ≥60% oocyte yield (%)	14 (45.2%)	22 (71.0%)	25.8% (2.1%, 49.5%, *P* = 0.042)	36 (58.1%)
Number of oocytes	12 (7, 18)	13 (10, 17)		12 (8, 17)
Number of mature (M2) oocytes	10 (5, 12)	10 (6, 14)		10 (6, 14)
Oocyte maturation rate as percentage^a^	83.0% (67.0%, 92.0%)	80.0% (67.0%, 92.0%)		82.0% (67.0%, 92.0%)
Oocyte yield as percentage^b^	52.9% (25.0%, 75.0%)	63.6% (40.0%, 75.0%)		58.1% (37.5%, 75.0%)
Number of patients with ≥4 eggs collected (%)	28 (90.3%)	31 (100%)		59 (95.2%)
Fertilization rate as percentage^c^	78.9% (55.6%, 83.3%)	78.6% (66.7%, 92.9%)		78.6% (64.2%, 88.9%)
Number of two pronuclear (2PN) zygotes	6 (3, 9)	8 (5, 11)		7.5 (4, 10)
Number of patients with at least one two pronuclear (2PN) zygote	30 (96.8%)	31 (100%)		61 (98.4%)
Number of cleaved embryos at Day 3 post ICSI	6 (3, 9)	8 (5, 10)		7 (4,10)
Number of embryos at Day 3 graded as 623 or 632	6 (3, 8)	6 (3, 9)		6 (3,9)
Number of patients with segmentation	1 (3.2%)	0 (0.0%)		1 (1.6%)
Number of patients with embryo transfer	29 (93.5%)	31 (100%)		60 (96.8%)
Number of embryos at Day 5	6 (3, 9)	6 (4, 9)		6 (3, 9)
Number of high quality blastocysts (≥3AB or ≥3BA) at Day 5	1 (0, 2)	1 (0, 3)		1 (0, 3)
Number of patients with Day 5 embryo transfer	26 (83.9%)	29 (93.5%)		55 (88.7%)
Number of patients with transfer of high quality embryo at Day 5	17 (54.8%)	17 (54.8%)		34 (54.8%)
Biochemical pregnancy rate per protocol (%)	10 (32.3%)	16 (51.6%)		26 (41.9%)
Clinical pregnancy rate per protocol (%)	7 (23.0%)	12 (39.0%)	16.0% (−0.08, 0.38, *P* = 0.199)	19 (30.6%)
Implantation rate (SD)^d^	23.3% (43.0)	37.0% (48.2)		30.3% (45.9)
Live birth rate per protocol (%)	6 (19.4%)	12 (39.0%)		18 (29.0%)

Contains medians (interquartile range) for continuous variables and totals (percentages of *N*) for categorical variables. AD = absolute difference; CI = confidence interval; *P* = *P* value. Single = patients receiving a single injection of kisspeptin. Double = patients receiving a second injection of kisspeptin 10 h after the first. Both = data for both single and double groups combined.

Primary outcome: proportion of patients achieving satisfactory oocyte maturation; defined as the proportion of patients achieving an oocyte yield ≥60%. Secondary outcomes: Implantation rate, occurrence of OHSS and reproductive hormone levels. Statistical comparison was only performed on the primary and secondary outcomes.

^a^Oocyte maturation rate is the percentage of oocytes collected which were mature.

^b^Oocyte yield is the percentage of mature oocytes collected from the number of follicles ≥14 mm in diameter on the final ultrasound scan prior to kisspeptin-54 trigger administration.

^c^Fertilization rate is the percentage of mature oocytes which fertilize to form two pronuclear (2PN) zygotes following ICSI.

^d^Implantation rate is defined as the percentage of embryos transferred which implant on assessment by ultrasound at 6 weeks of gestation.

Biochemical pregnancy rate is defined as serum βhCG > 10 iU/L 11 days after embryo transfer Clinical pregnancy rate is defined as intrauterine gestational sac with heartbeat on ultrasound at 6 weeks gestation Live birth rate is defined as the number of protocols undertaken that resulted in a live birth.

### Primary outcome: oocyte yield

The proportion of patients achieving an oocyte yield ≥60% was improved from 45% in the single kisspeptin-54 group to 71% in the double kisspeptin-54 group (*P* = 0.042). The absolute risk difference in the proportion of patients achieving an oocyte yield ≥60% between the single and double groups was + 26% (CI 2–50%). The odds of achieving an oocyte yield ≥60% in the double group was increased 2.97-fold (CI 1·04–8.48) compared with the single group.

### Secondary outcomes

#### Effect of a second dose of kisspeptin-54 administration on reproductive hormone levels

**Figure 3:**
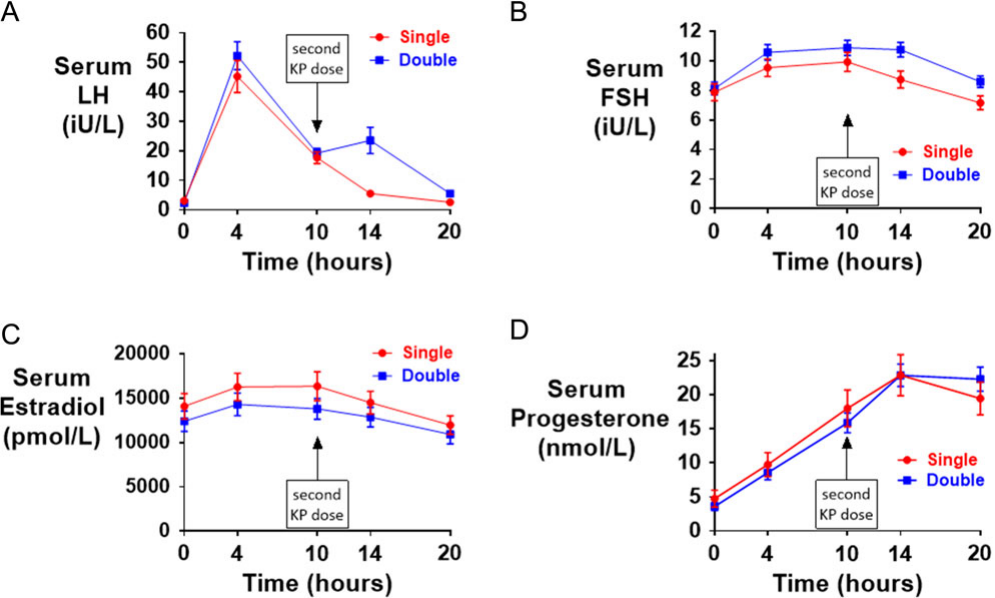
Reproductive hormonal response following kisspeptin-54 trigger administration. All patients received 9.6 nmol/kg of kisspeptin-54 subcutaneously 36 h prior to oocyte retrieval to trigger oocyte maturation. Patients were then randomized to receive a second injection 10 h later of either saline (Single shown in red) or kisspeptin-54 9.6 nmol/kg (Double shown in blue). Serum LH in iU/L ( **A**), serum FSH in iU/L (**B**), serum estradiol in pmol/L ( **C**) and serum progesterone in nmol/L (**D**) is presented by kisspeptin-54 dosing group (single or double).

As expected, the first injection of kisspeptin-54 led to a similar rise in LH and FSH between the single and double groups. Patients receiving kisspeptin-54 as their second injection (double group) had higher serum LH and FSH levels at 4 and 10 h after the second injection, when compared with patients receiving saline as their second injection (single group). The mean change in serum LH was increased 3.3-fold in the single group and 14.7-fold in the double group at 4 h following the second injection when compared with pre-trigger levels (see Fig. 3 and Supplemental Table S1 for serum hormonal profiles by kisspeptin-54 dosing group). Serum LH continued to fall during the 4 h following the second injection by 12.1 ± 7.5 iU/L in the Single group, but rose by 4.3 ± 24.9 iU/L in the Double group.

Interestingly, in patients randomized to a double dose of kisspeptin-54, those with a lower serum LH-level just prior to the second dose of kisspeptin-54 had a greater subsequent LH-response and vice versa (see ‘Supplemental Figure S2’ for bar-chart of change in serum LH at 4 h following the second dose of kisspeptin-54 by categories of serum LH just prior to administration of the second dose of kisspeptin-54).

#### Pregnancy

Implantation rates were 23.3% following a single dose and 37.1% following a double dose of kisspeptin-54 (OR 2.1; CI 0.68-6.3). The live birth rate was 19.4% (6/31) in the single group and 39% (12/31) in the double group (see Table II for pregnancy outcomes).

#### Rates of OHSS

There was no difference in the occurrence of OHSS between those who received a single or double dose of kisspeptin-54. There was one moderate early OHSS in single group (1.6%), and one mild late OHSS (1.6%) in the double group. The woman diagnosed with moderate early OHSS was admitted for analgesia for <24 h the day following oocyte retrieval and although she required no medical intervention, she was treated with segmentation in accordance with the protocol (see ‘Supplementary Table SII’ for full data on OHSS screening).

### Adverse events

Kisspeptin-54 was well-tolerated in all 62 women in this study. One woman in the single group had an unexplained second trimester miscarriage at 19 weeks of gestation.

## Discussion

This study shows that patients receiving two doses of kisspeptin-54 to trigger oocyte maturation had an increased duration of LH and FSH exposure compared to patients receiving a single dose. This was associated with those receiving two doses of kisspeptin-54 having an increased proportion achieving an oocyte yield ≥60%. Despite the study population being at high risk of developing OHSS, a second dose of kisspeptin-54 did not increase the occurrence of excessive ovarian response or OHSS.

For this trial, we chose a patient-centric primary outcome, namely the proportion of patients achieving an oocyte yield ≥60%. This was chosen as a thresh-hold for trigger efficacy that would increase the chance of progression to subsequent stages of IVF treatment such as embryo transfer. All patients who achieved an oocyte yield ≥60% had an embryo available for transfer and 25/36 (69%) had a high quality blastocyst available for transfer. By contrast, 1 of 26 (3.8%) patients with an oocyte yield ≤60% did not have an embryo available for transfer and 10 of 26 (38%) patients had a high quality embryo available for transfer. Thus, from a patient’ perspective this primary outcome represents a clinically meaningful measure of trigger efficacy.

In order to investigate the safety and efficacy of kisspeptin-54 in women at high risk of developing OHSS, the inclusion criteria for this trial were a high serum AMH ≥40 pmol/L or AFC ≥23. The women in this study had a mean AFC of 40 and mean serum AMH of 52 pmol/L confirming that we had successfully recruited women at high risk of developing OHSS during IVF treatment (Lee *et al.*, 2008; Jayaprakasan *et al.*, 2012). Once a woman begins controlled ovarian stimulation, the number of follicles ≥11 mm prior to trigger can be used to further delineate the risk of OHSS (Papanikolaou *et al.*, 2006). The mean number of follicles ≥11 mm on the morning of trigger administration was 24 in this trial. Some authors recommend that all women with 25 or more follicles ≥11 mm have segmentation following current triggers of oocyte maturation to avoid late-onset OHSS (Humaidan *et al.*, 2013). However, a universal segmentation policy is often not favoured by patients, as it may result in increased cost of treatment and a longer time to pregnancy by ~2–3 months when compared with fresh embryo transfer. Despite 48% (30/62) of patients in our trial having more than 25 follicles ≥11 mm on the day of trigger, almost all patients (61/62; 98.4%) were still able to safely receive fresh embryo transfer following kisspeptin-54 triggering.

It seemed plausible that prolonging the duration of the LH-surge with a second dose of kisspeptin-54 could induce OHSS in the high risk patient, as described in case-reports following GnRH-agonist triggering (Fatemi *et al.*, 2014; Gurbuz *et al.*, 2014; Ling *et al.*, 2014). However, kisspeptin-54 has some unique actions suggesting that despite prolonging the duration of the LH-surge with a second dose of kisspeptin-54, it may still reduce the incidence of OHSS due to: (i) its shorter duration of action compared to hCG (Abbara *et al.*, 2015; Thomsen and Humaidan, 2015); (ii) its action to release a pre-formed endogenous pool of GnRH from the hypothalamus (Dhillo *et al.*, 2005; Jayasena *et al.*, 2015) and as the quantity of endogenous GnRH is individually determined, kisspeptin-54 is less likely to produce an excessive ovarian response sufficient to prompt OHSS (Abbara *et al.*, 2015); (iii) kisspeptin-54 directly inhibits the production of the predominant mediator of OHSS, vascular endothelial growth factor (VEGF), from the ovaries following controlled ovarian stimulation (Cho *et al.*, 2009). Hence, it is conceivable that a second dose of kisspeptin-54 could improve oocyte yield by prolonging the LH-surge, but without additionally increasing the risk of OHSS. In keeping with this, there was no increase in mean or maximal ovarian diameter following a second dose of kisspeptin-54 (see Supplemental Table SII for OHSS screening data). In addition, only one woman from 62 patients at high risk of developing OHSS (from Single group) was treated with segmentation. She had an AFC of 84, a serum AMH of 165 pmol/L, developed 73 follicles ≥11 mm on the morning of trigger and had a serum estradiol of 38 264 pmol/L just prior to trigger administration. She had 46 oocytes retrieved, of which 41 were mature. She developed abdominal pain the day following oocyte retrieval and was thus admitted for analgesia and observation for <24 h before her symptom spontaneously settled without requiring any medical intervention. Prospective clinical trials in which routine screening for OHSS was conducted (Toftager *et al.*, 2016) often report an increased frequency of OHSS when compared with retrospective trials relying upon patient instigated reporting of OHSS (Serour *et al.*, 1998). Thus, it is reassuring that despite routine screening of women at high risk of OHSS in this study, two doses of kisspeptin-54 can safely be administered without causing an increased risk of ovarian hyper-response and OHSS.

Interestingly, this study revealed novel data delineating the pharmacodynamic impact of repeated dosing of kisspeptin-54. Patients receiving two doses of kisspeptin-54 had a variable LH-response following the second dose, which appeared to be resultant on the LH-response following the first kisspeptin-54 injection. Patients who had a substantial LH-rise following the first dose of kisspeptin-54 had a lower further rise following the second dose of kisspeptin-54. Conversely, those with a lower LH-response following the first dose of kisspeptin-54 had a more pronounced ‘rescue’ LH-rise following the second dose of kisspeptin-54 (see ‘Supplemental Figure S2’ for variation in response following the second dose of kisspeptin-54). Thus, a second dose of kisspeptin-54 was able to eliminate the retrieval of fewer than four oocytes from 9.7% in the single group to 0% in the double group. Moreover, it has been suggested that retrieving a greater number of oocytes beyond 15 does not yield further increases in live birth rates (van der Gaast *et al.*, 2006; Sunkara *et al.*, 2011). A second dose of kisspeptin-54 improved the proportion achieving a clinically effective oocyte yield, but importantly did not increase the proportion of patients having an excessive ovarian response (see ‘Supplemental Figure S1’ for histogram of number of oocytes retrieved by kisspeptin-54-dosing group). It is interesting to speculate as to why the variable response following the second dose of kisspeptin-54 was observed; this property of kisspeptin-54 pharmacodynamics could be explained by its unique mechanism of action. Kisspeptin-54 acts at the level of the hypothalamus to cause the release of a pre-formed endogenous pool of GnRH (Jayasena *et al.*, 2015). Patients with a greater LH-rise following the first dose of kisspeptin-54 may have depleted their hypothalamic GnRH stores such that a second injection of kisspeptin-54 resulted in a minimal further LH-response. In contrast, patients with a lower rise in LH following the first dose of kisspeptin-54 may have remaining hypothalamic GnRH stores, allowing a second dose of kisspeptin-54 to induce a rescue LH-response. A less likely possibility is that the variability in LH-response reflects tachyphylaxis at the kisspeptin-54 receptor; tachyphylaxis is more usually associated with high-dose frequent administration of kisspeptin-54 rather than two intermediate doses administered 10 h apart as in this study (Abbara *et al.*, 2013). This ability of kisspeptin-54 to stimulate gonadotropin secretion in response to a patient's endogenous GnRH reserve, without causing excessive response is of particular value in women undergoing IVF treatment at high risk of developing OHSS in individualizing the LH-response achieved following the second dose of kisspeptin-54.

A limitation of this trial is that we only examined one dose of kisspeptin-54 (9.6 nmol/kg) in the single and double groups. Furthermore, only one dosing interval of 10 h was evaluated and it is possible that the full benefit on oocyte maturation of the second dose of kisspeptin-54 may not yet have been fully attained, as oocyte retrieval was routinely performed only 26 h after the second injection. Therefore, it remains possible that other regimens utilizing different kisspeptin-54 dosages and other dosing intervals could further improve oocyte maturation in individual patients. The study was designed to detect a difference in oocyte maturation, but was not powered to detect a difference in implantation rates. Thus, further larger studies are now required to directly compare kisspeptin triggering against other currently used triggers of oocyte maturation.

In summary, we observe that a second dose of kisspeptin-54 administered 10 h following the first can improve the proportion of patients achieving an oocyte yield ≥60%, but without increasing the risk of ovarian overstimulation and OHSS. Thus, a second dose of kisspeptin-54 is a safe option in patients undergoing IVF treatment to further optimize oocyte maturation, even those at high risk of OHSS.

## Authors’ roles

All authors provided contributions to study conception and design, acquisition of data or analysis and interpretation of data, drafting the article or revising it critically for important intellectual content, and final approval of the version to be published. Here are the most important contributions of each author: A.A., S.R.B., G.H.T. and WSD designed the study. Data was collected by A.A., S.C., R.I., J.P., A.C., S.N., D.P., R.R., C.I.-E., R.R., A.N. and S.V. OHSS scoring was independently carried out by R.S. and S.L. Analysis was carried out by L.H. W.S.D. takes final responsibility for this article.

## Funding

This paper presents independent research funded by grants from the Medical Research Council (MRC), Biotechnology and Bioscience Research Council (BBSRC) and National Institute for Health Research (NIHR) and supported by the NIHR/WellcomeTrust Imperial Clinical Research Facility and Imperial Biomedical Research Centre. The views expressed are those of the author(s) and not necessarily those of the MRC, BBSRC, the NHS, the NIHR or the Department of Health. The section of Endocrinology and Investigative Medicine is funded by grants from the MRC, BBSRC, NIHR and is supported by the NIHR Biomedical Research Centre Funding Scheme. A.A. and A.N.C. are supported by National Institute of Health Research (NIHR) Clinical Lectureships. S.C. is supported by funding from an NIHR Research Professorship. J.K.P. is supported by NIHR Academic Clinical Fellowship and Imperial College Healthcare NHS trust Charity Fellowship. C.I.-E and R.R. are supported by an MRC Clinical Training Fellowship. W.S.D. is supported by an NIHR Research Professorship (grant reference RP-2014-05-001).

## Conflict of interest

None declared.

## Supplementary Material

Supplementary Figure SI

Supplementary Figure SII

Supplementary Methods

Supplementary Table SI

Supplementary Table SII
