# Supplementary material for: A second dose of kisspeptin-54 improves oocyte maturation in women at high risk of ovarian hyperstimulation syndrome: a Phase 2 randomized controlled trial
Source: Hum Reprod. 2017 Aug 8;32(9):1915–24. doi: 10.1093/humrep/dex253 (PMC5850304; doi:10.1093/humrep/dex253)
Supplement: Supplementary Figure SI [file dex253supplementalfigures1.pdf]

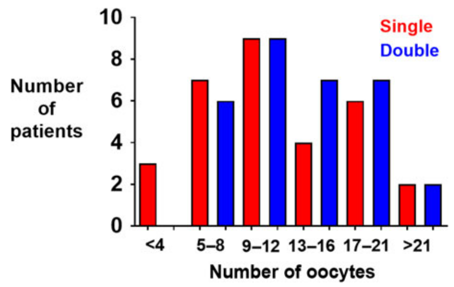

Supplemental Figure S1 Histogram of number of oocytes retrieved by kisspeptin dosing group Frequency of patients by categories of number of oocytes retrieved is presented for kisspeptin dosing groups: Number of patients receiving a single dose of kisspeptin is shown in red bars ($n = 31$) and number of patients receiving two doses of kisspeptin (double) shown in blue bars ($n = 31$).
