# Supplementary material for: A second dose of kisspeptin-54 improves oocyte maturation in women at high risk of ovarian hyperstimulation syndrome: a Phase 2 randomized controlled trial
Source: Hum Reprod. 2017 Aug 8;32(9):1915–24. doi: 10.1093/humrep/dex253 (PMC5850304; doi:10.1093/humrep/dex253)
Supplement: Supplementary Figure SII [file dex253supplementalfigures2.pdf]

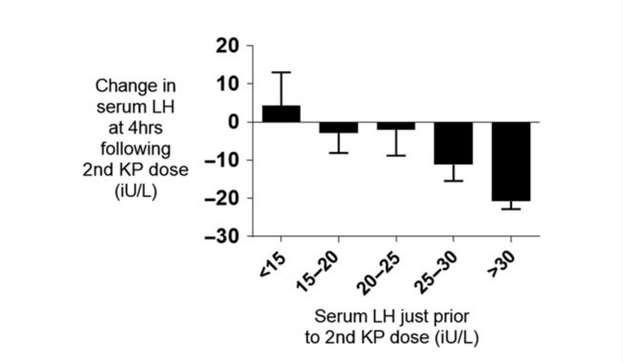

Supplemental Figure S2 Change in serum LH following the second dose in patients randomized to double dose of kisspeptin (KP). Median (IQR) change in serum LH from just prior to the second dose of kisspeptin to 4 h thereafter is shown for patients randomized to two doses of kisspeptin (double; $n = 31$) by categories of serum LH just prior to the second kisspeptin dose being administered. A greater subsequent response to the second dose of kisspeptin is observed in patients with a lower serum LH just prior to its administration.
