# Supplementary material for: A second dose of kisspeptin-54 improves oocyte maturation in women at high risk of ovarian hyperstimulation syndrome: a Phase 2 randomized controlled trial
Source: Hum Reprod. 2017 Aug 8;32(9):1915–24. doi: 10.1093/humrep/dex253 (PMC5850304; doi:10.1093/humrep/dex253)
Supplement: Supplementary Methods [file dex253supplementalmethods.pdf]

**Human Reproduction, 2017**
doi:10.1093/humrep/dex253



# SUPPLEMENTARY DATA

## Supplemental methods

*Further detail of study protocol*

From Day 2 or 3 of the menstrual cycle, patients received daily SC recombinant FSH (recFSH) injections (Gonal F 112.5 iU, Merck Serono, Geneva, Switzerland, administered daily at midday); pelvic ultrasound scans were performed 5 days after commencing recFSH, to determine ovarian follicular development and appropriately titrate the dose of recFSH. Daily subcutaneous GnRH antagonist injections (Cetrotide 0.25 mg, Merck Serono, injected at 9 p.m. daily) were commenced from Day 5 of recFSH injections until 24 h prior to kisspeptin trigger injection. If serum LH was undetectable (<0.5 iU/L) on Day 7 of recFSH injections, then the dose of cetrotide was halved to 0.125 mg daily. When at least three ovarian follicles ≥18 mm diameter were visible on ultrasound, all patients received a subcutaneous bolus injection of kisspeptin administered by a study investigator to trigger oocyte maturation at 36 h prior to oocyte retrieval (between 20:30 and 23:00 h). The dose of kisspeptin was weight-adjusted (9.6 nmol/kg), and injection volumes ranged from a minimum of 300 –580 µl. Patients were then randomized to receive either a second dose of kisspeptin (9.6 nmol/kg), or saline placebo injection at 10 h following the first kisspeptin trigger injection. Patients, IVF physicians and embryologists were blinded to the randomization.

Transvaginal ultrasound-directed oocyte retrieval (TVOR) was carried out 36 h following kisspeptin administration under intravenous anaesthesia (propofol). ICSI was performed in all study cycles using sperm from the male partner to allow for assessment of oocyte maturation.

Definitions of other clinical outcomes reported include fertilization rate (percentage of mature oocytes which fertilized to form two pronuclear (2PN) zygotes following ICSI), embryo formation and quality, biochemical pregnancy rate (serum βhCG > 10iU/L 11 days after embryo transfer) and clinical pregnancy rate (intrauterine gestational sac with heartbeat on ultrasound at 6 weeks gestation).

*Assessment of ovarian hyperstimulation syndrome (OHSS)*

Women were screened by symptoms (abdominal pain, abdominal bloating, diarrhoea, nausea, vomiting, subjective reduction in urine output), blood analysis (hemaglobin, hematocrit, white cell count, liver function, renal function, coagulation profile) and ultrasound parameters (ovarian size, presence of free fluid in pouch of douglas, adnexae, abdomen or pleural cavity). OHSS was graded according to the criteria of Golan *et al.* 1989 with updated categorization by Navot *et al.* (1992) (Golan *et al.*, (1989); Navot *et al.*, 1992; Abbara *et al.*, 2015). OHSS was independently graded by two experienced IVF clinicians external to the study team (S.L. and R.S.), who were provided with blinded data concerning patient symptom, blood analysis and ultrasound parameters. In the event of any discrepancy in categorization of OHSS, the more severe classification was used. Women who did not have fresh embryo transfer due to lack of oocyte maturation/ fertilization were screened by symptoms alone due to low risk of OHSS. In order to safeguard participant well-being throughout the study, a clinical decision was requested from the senior IVF study clinician (G.T.) prior to kisspeptin trigger administration in all patients with ≥18 follicles ≥11 mm, or serum estradiol level ≥18 000 pmol/L, as to whether to proceed with fresh embryo transfer, or to cryopreserve all embryos and conduct embryo transfer in a subsequent cycle (segmentation). Women who had segmentation were screened for early OHSS at 5 days following oocyte retrieval, but were screened for late OHSS by symptoms alone unless the results of early screening revealed any abnormality on ultrasound or blood parameters.

*Embryo grading*

All embryos were graded at Day 3 by an embryologist, using the British Fertility Society (BFS) and Association of Clinical Embryologist (ACE) embryo grading scheme for cleavage stage embryos, which describes embryos based on cell number, blastomere size and fragmentation. If at Day 3 at least two embryos had six or more cells, <20% difference in blastomere diameter and <20% fragmentation, they were incubated until Day 5 post oocyte retrieval in order that the strongest embryos could be identified for transfer. On Day 5, embryos were graded for blastocyst expansion (1–6), inner cell mass (A–E) and trophectoderm (A–C). At blastocyst stage, embryos which scored at least 3 for blastocyst expansion and A or B for inner cell mass and trophectoderm were classified as 'high quality embryos'.

*Peptide synthesis*

Kisspeptin-54 was synthesized and purified by Bachem (Bachem Holding AG, Bubendorf. Switzerland). Sterile vials of Kisspeptin-54 were produced by the Clinalfa brand of Bachem (Bachem Distribution Services GmbH, Weil am Rhein, Germany). Both productions were prepared according to Good Manufacturing Practice (GMP). Vials of freeze-dried kisspeptin-54 were stored at –20°C and reconstituted in 0.9% saline as previously described (Abbara *et al.*, 2015).

*Hormonal assay methodology*

Serum LH, FSH, oestradiol and progesterone were measured using automated chemiluminescent immunoassays (Abbott Diagnostics, Maidenhead, UK). Interassay coefficients of variation were as follows: LH, 3.4%; FSH, 3.5%; oestradiol, 3.4%; progesterone, 1.8%. Limits of detectability for each assay were as follows: LH 0.07mIU/ml; FSH 0.05mIU/ml; oestradiol 70 pmol/l (19 pg/mL); progesterone 0.3 nmol/L (0.1 ng/mL).

AMH was measured using an electrochemiluminescence immunoassay (Roche Cobas e411 assay, Switzerland). The analytical range of detection was 0·01–23 ng/ml (0·071–164.2 pmol/L). The lower limit of detection is 0.01 ng/ml (0.071 pmol/L) and the limit of quantitation = 0.030 ng/mL. The interassay coefficient of variance is between 2.9% and 4.4%: 4.4% at 0.232 ng/ml (1.66 pmol/L) and 3.8% at the higher end of 18.8 ng/ml (134.23 pmol/L). The intraassay coefficient of variance is between 1.1% and 1.8%: 1.8% at 0.232 ng/ml (1.66 pmol/L) and 1.5% at the higher end of 18.8 ng/ml (134.23 pmol/L).
