# Supplementary material for: A second dose of kisspeptin-54 improves oocyte maturation in women at high risk of ovarian hyperstimulation syndrome: a Phase 2 randomized controlled trial
Source: Hum Reprod. 2017 Aug 8;32(9):1915–24. doi: 10.1093/humrep/dex253 (PMC5850304; doi:10.1093/humrep/dex253)
Supplement: Supplementary Table SI [file dex253supplementarytablesi.pdf]

Supplementary Table SI **Reproductive hormone levels in patients receiving one or two injections of kisspeptin-54.**

Time point	LH (iU/L)			FSH (iU/L)			Estradiol (pmol/L)		Progesterone (nmol/L)	
	Single	Double	*P* value	Single	Double	*P* value	Single	Double	Single	Double
Pre-first KP injection	3.1 (3.7)	2.3 (1.4)		7.9 (3.3)	8.1 (2.5)		14 085 (8042)	12 402 (6456)	4.7 (6.9)	3.6 (4.4)
Four hours post first KP injection	45.2 (30.4)	52.2 (26.6)		9.6 (3.3)	10.6 (2.9)		16 272 (8651)	14 302 (7119)	9.7 (9.7)	8.6 (5.7)
Ten hours post first KP injection (pre second injection)	17.7 (11.1)	19.2 (8.9)		9.9 (3.6)	10.9 (2.8)		16 364 (9144)	13 809 (6482)	18.0 (15.1)	15.9 (8.0)
Four hours post second KP injection	5.5 (3.8)	23.5 (24.9)	0.0002	8.8 (3.2)	10.8 (2.6)	0.01	14 502 (7146)	12 880 (6148)	22.9 (16.8)	22.8 (9.3)
Ten hours post second KP injection	2.6 (1.8)	5.5 (5.4)		7.2 (2.6)	8.6 (2.2)		11 982 (5922)	10 927 (5972)	19.5 (13.4)	22.3 (9.8)

Mean (SD) reproductive hormone levels (serum LH in iU/L, serum FSH in iU/L, serum estradiol in pmol/L, serum progesterone in nmol/L) in patients receiving either a single injection of kisspeptin-54 9.6 nmol/kg (Single, $n = 31$), or two injections of kisspeptin-54 at 9.6 nmol/kg at a 10 h interval (Double, $n = 31$) to trigger oocyte maturation. A two way unpaired *t*-test was used to compare absolute serum LH levels, and absolute serum FSH levels at 4 h following the second injection (saline in Single group, or kisspeptin-54 at 9.6 nmol/kg in Double group) between the two groups (secondary analysis).

References

Abbara A, Jayasena CN, Christopoulos G, Narayanaswamy S, Izzi-Engbeaya C, Nijher GMK, Comninos AN, Peters D, Buckley A, Ratnasabapathy R *et al.* Efficacy of kisspeptin-54 to trigger oocyte maturation in women at high risk of ovarian hyperstimulation syndrome (OHSS) during in vitro fertilization (IVF) therapy. *J Clin Endocrinol Metab* 2015;**100**:3322–3331.

Golan A, Ron-El R, Herman A, Soffer Y, Weinraub Z, Caspi E. Ovarian hyperstimulation syndrome: an update review. *Obstet Gynecol Surv* 1989;**44**:430–440.

Navot D, Bergh PA, Laufer N. Ovarian hyperstimulation syndrome in novel reproductive technologies: prevention and treatment. *Fertil Steril* 1992;**58**:249–261.
