# Supplementary material for: A second dose of kisspeptin-54 improves oocyte maturation in women at high risk of ovarian hyperstimulation syndrome: a Phase 2 randomized controlled trial
Source: Hum Reprod. 2017 Aug 8;32(9):1915–24. doi: 10.1093/humrep/dex253 (PMC5850304; doi:10.1093/humrep/dex253)
Supplement: Supplementary Table SII [file dex253supplementarytablesii.pdf]

**Supplementary Table SII Ovarian hyperstimulation syndrome (OHSS) in high risk women following kisspeptin-54 trigger.**

| *OHSS screening* | Early OHSS | | | | Late OHSS | | | |
|---|---|---|---|---|---|---|---|---|
| *Kisspeptin dosing group* | Single | Double | All | AD (CI) *P* value | Single | Double | All | AD (CI) *P* value |
| *Number of patients diagnosed with OHSS*[¥] | | | | | | | | |
| Normal | 30 (96.8%) | 31 (100%) | 61 (98.4%) | | 31 (100%) | 30 (96.8%) | 61 (98.4%) | |
| Mild | 0 | 0 | 0 | | 0 | 1 (3.2%) | 1 (1.6%) | |
| Moderate | 1 (3.2%) | 0 | 1 (1.6%) | | 0 | 0 | 0 | |
| Severe | 0 | 0 | 0 | | 0 | 0 | 0 | |
| Critical | 0 | 0 | 0 | | 0 | 0 | 0 | |
| *OHSS symptoms* | | | | | | | | |
| Number of patients screened by OHSS symptoms (%) | 31 (100%) | 31 (100%) | 62 (100%) | | 31 (100%) | 31 (100%) | 62 (100%) | |
| Number of patients with ≥1 symptom potentially consistent with OHSS (%) | 9 (29.0%) | 7 (22.6%) | 16 (25.8%) | | 9 (29.0%) | 9 (29.0%) | 18 (29.0%) | |
| Number of patients requiring medical intervention for OHSS (%) | 0 (0.0%) | 0 (0.0%) | 0 (0.0%) | | 0 (0.0%) | 0 (0.0%) | 0 (0.0%) | |
| Number of patients requiring hospitalization for OHSS (%) | 1 (3.2%) | 0 (0.0%) | 1 (1.6%) | | 0 (0.0%) | 0 (0.0%) | 0 (0.0%) | |
| *Sonographic screening* | | | | | | | | |
| Number of patients screened with pelvic ultrasound (%) | 31 (100%) | 30 (96.8%) | 61 (98.4%) | | 30 (96.8%) | 31 (100%) | 61 (98.4%) | |
| Mean ovarian volume in ml (SD) | 68.6 (52.1) | 48.2 (28.9) | 58.6 (43.2) | –20.4 (–39.3, –1.5) $P = 0.034$ | 28.9 (40.9) | 16.1 (15.9) | 22.4 (31.3) | –12.8 (–22.5, –3.3) $P = 0.006$ |
| Mean ovarian diameter in mm (SD) | 41.5 (10.1) | 38.7 (7.9) | 40.2 (9.1) | | 29.6 (9.9) | 25.9 (6.0) | 27.8 (8.3) | |
| Maximal ovarian diameter in mm (SD) | 64.2 (15.1) | 58.1 (11.0) | 61.3 (13.6) | –6.1 (–12.9, 0.60) $P = 0.079$ | 44.7 (14.0) | 42.4 (9.5) | 43.5 (11.9) | –2.3 (–7.7, 3.1) $P = 0.410$ |
| Number of patients with maximum ovarian diameter ≥5 cm (%) | 24 (77.4%) | 22 (73.3%) | 46 (75.4%) | | 7 (23.3%) | 7 (22.6%) | 14 (23.0%) | |
| Number of patients with maximum ovarian diameter ≥8 cm (%) | 5 (16.1%) | 1 (3.3%) | 6 (9.8%) | | 1 (3.3%) | 0 (0.0%) | 1 (1.6%) | |
| Number of patients with maximum ovarian diameter ≥12 cm (%) | 0 (0.0%) | 0 (0.0%) | 0 (0.0%) | | 0 (0.0%) | 0 (0.0%) | 0 (0.0%) | |
| Total free fluid in abdomen, POD/Adnexa in ml (SD) | 5.8 (8.1) | 3.4 (5.8) | 4.6 (7.1) | | 3.0 (11.8) | 0.8 (2.3) | 1.9 (8.4) | |
| Number of patients with ≥ Grade 2 ascites (%) | 0 (0.0%) | 0 (0.0%) | 0 (0.0%) | | 0 (0.0%) | 0 (0.0%) | 0 (0.0%) | |
| Number of patients with pleural effusion (%) | 0 (0.0%) | 0 (0.0%) | 0 (0.0%) | | 0 (0.0%) | 0 (0.0%) | 0 (0.0%) | |
| *Blood parameters* | | | | | | | | |
| Number of patients screened with Blood analysis (%) | 31 (100%) | 29 (93.5%) | 60 (96.8%) | | 30 (96.8%) | 31 (100%) | 61 (98.4%) | |
| Number of patients with haematocrit >45% (%) | 0 (0.0%) | 0 (0.0%) | 0 (0.0%) | | 0 (0.0%) | 0 (0.0%) | 0 (0.0%) | |
| Number of patients with serum sodium ≥132 mmol/L (%) | 0 (0.0%) | 0 (0.0%) | 0 (0.0%) | | 0 (0.0%) | 0 (0.0%) | 0 (0.0%) | |
| Number of patients with serum potassium ≥5.0 mmol/L (%) | 0 (0.0%) | 0 (0.0%) | 0 (0.0%) | | 0 (0.0%) | 0 (0.0%) | 0 (0.0%) | |
| Number of patients with ALT ≥2 × ULN (%) | 3 (9.7%) | 1 (3.4%) | 4 (6.7%) | | 1 (3.3%) | 1 (3.2%) | 2 (3.4%) | |

*Continued*

**Supplementary Table SII** *Continued*

| *OHSS screening* | Early OHSS | | | | Late OHSS | | | |
|---|---|---|---|---|---|---|---|---|
| *Kisspeptin dosing group* | Single | Double | All | AD (CI) *P* value | Single | Double | All | AD (CI) *P* value |
| Number of patients with AST ≥2 × ULN (%) | 2 (6.5%) | 1 (3.4%) | 3 (5.0%) | | 0 (0.0%) | 0 (0.0%) | 0 (0.0%) | |
| Number of patients with total protein ≥80 g/L (%) | 1 (3.2%) | 0 (0.0%) | 1 (1.7%) | | 0 (0.0%) | 0 (0.0%) | 0 (0.0%) | |
| Number of patient with creatinine ≥110 µmol/L (%) | 0 (0.0%) | 0 (0.0%) | 0 (0.0%) | | 1 (3.3%) | 0 (0.0%) | 1 (1.6%) | |

Mean (SD) is presented for continuous variables and number of patients (%) for categorical variables.
[¥]Diagnosis of OHSS was performed by two experienced IVF physicians (R.S. and S.L.) independent of the study team provided with blinded data according to the criteria of Golan *et al.* (1989) with updated categorization of severe and critical OHSS by Navot *et al.* (1992).
AD, absolute difference; CI, confidence interval; POD, pouch of douglas; ALT, alanine transaminase; AST, aspartate transaminase; ULN, upper limit of normal.

## Reference


Navot D, Bergh PA, Laufer N. Ovarian hyperstimulation syndrome in novel reproductive technologies: prevention and treatment. *Fertil Steril* 1992;**58**:249–261.
